# Understanding and optimising patient and public involvement in trial oversight: an ethnographic study of eight clinical trials

**DOI:** 10.1186/s13063-020-04495-9

**Published:** 2020-06-18

**Authors:** K. D. Coulman, A. Nicholson, A. Shaw, A. Daykin, L. E. Selman, R. Macefield, G. W. Shorter, H. Cramer, M. R. Sydes, C. Gamble, M. E. Pick, G. Taylor, J. A. Lane

**Affiliations:** 1 MRC ConDuCT-II Hub for Trials Methodology Research, Population Health Sciences, Bristol Medical School, University of Bristol, Bristol, BS8 2PS UK; 2 Centre for Improving Health Related Quality of Life, School of Psychology, Queen’s University Belfast, Belfast, BT9 5BN UK; 3 MRC Clinical Trials Unit at UCL, Institute of Clinical Trials and Methodology, University College London, London, WC1J 6JL UK; 4 MRC London Hub for Trial Methodology Research, London, UK; 5 MRC North West Hub for Trials Methodology Research, Institute of Translational Medicine, University of Liverpool, Liverpool, L69 3BX UK; 6 Bristol Randomised Trials Collaboration, Population Health Sciences, Bristol Medical School, University of Bristol, Bristol, BS8 2PS UK

**Keywords:** Public involvement, User involvement, Patient involvement, Randomised trials, Trial steering committees, Trial management groups, Trial monitoring, Trial oversight

## Abstract

**Background:**

Trial oversight is important for trial governance and conduct. Patients and/or lay members of the public are increasingly included in trial oversight committees, influenced by international patient and public involvement (PPI) initiatives to improve the quality and relevance of research. However, there is a lack of guidance on how to undertake PPI in trial oversight and tokenistic PPI remains an issue. This paper explores how PPI functions in existing trial oversight committees and provides recommendations to optimise PPI in future trials. This was part of a larger study investigating the role and function of oversight committees in trials facing challenges.

**Methods:**

Using an ethnographic study design, we observed oversight meetings of eight UK trials and conducted semi-structured interviews with members of their trial steering committees (TSCs) and trial management groups (TMGs) including public contributors, trial sponsors and funders. Thematic analysis of data was undertaken, with findings integrated to provide a multi-perspective account of how PPI functions in trial oversight.

**Results:**

Eight TSC and six TMG meetings from eight trials were observed, and 66 semi-structured interviews conducted with 52 purposively sampled oversight group members, including three public contributors. PPI was reported as beneficial in trial oversight, with public members contributing a patient voice and fulfilling a patient advocacy role. However, public contributors were not always active at oversight meetings and were sometimes felt to have a tokenistic role, with trialists reporting a lack of understanding of how to undertake PPI in trial oversight. To optimise PPI in trial oversight, the following areas were highlighted: the importance of planning effective strategies to recruit public contributors; considering the level of oversight and stage(s) of trial to include PPI; support for public contributors by the trial team between and during oversight meetings.

**Conclusions:**

We present evidence-based recommendations to inform future PPI in trial oversight. Consideration should be given at trial design stage on how to recruit and involve public contributors within trial oversight, as well as support and mentorship for both public contributors and trialists (in how to undertake PPI effectively). Findings from this study further strengthen the evidence base on facilitating meaningful PPI within clinical trials.

## Introduction

Robust clinical trial oversight helps to safeguard participants by ensuring that trial conduct is in accordance with international Good Clinical Practice guidelines, and trial results are accurately reported [1, 2]. The UK Medical Research Council (MRC) distinguishes between three levels of trial oversight: the trial steering committee (TSC), which provides overall supervision for the trial and is comprised of an independent Chair and a majority of independent members; the independent data monitoring committee (iDMC) responsible for considering emerging trial data in relation to participant safety; and the trial management group (TMG), comprised of the trial team which facilitates the day-to-day running of the trial [3].

Lay members of the public or patients with a relevant clinical condition are being included more frequently in TSCs and TMGs [4, 5], based on the recognition of the potential benefits of involving patients and the public in healthcare research [6–9]. Incorporating the priorities of healthcare users into research has the potential to improve the relevance of research and reduce research “waste” [9, 10]. There is also a moral imperative to patient and public involvement (PPI) in research based on the belief that healthcare users should be involved in decisions about research and healthcare that will affect them [11]. The National Institute for Health Research (NIHR) National Standards for Public Involvement highlight the importance of involving the public in research governance and leadership to ensure “decisions promote and protect the public interest” [12]. Both the MRC and the NIHR, two major funders of UK clinical trials, recommend that TSCs include patient or public member(s), although no such guidance exists for TMGs [3, 13]. PPI can occur from initial idea development through to dissemination of trial results [14–16] and can be especially beneficial for shaping trial design and the selection of outcomes relevant to patients [8, 17]. PPI has also been reported to improve the recruitment of participants into trials [18]. However, not all researchers are clear about the benefits of PPI, and the risk of tokenistic PPI or lack of meaningful partnership is a problem [5, 19–24].

Previous research has highlighted a lack of clarity and guidance about the role and impact of PPI in trial oversight [4, 5, 25, 26] and conduct [20]. A previous qualitative study found the inclusion of public contributors on TMGs rather than TSCs was perceived as more useful as the TMG meet more frequently and offer more opportunities for involvement [5]. However, a limitation of this study was the reliance on retrospective interviews, which are subject to recall bias, and trials were funded some time ago (2006–2010) which does not reflect recent emphasis on co-production and the involvement of PPI [4]. Limited qualitative research investigating the impact of PPI have not focused on trials specifically [27], been limited to a single trials unit [28], or limited to one type of trial (surgical trials) [22], and none have used an ethnographic approach to observe actual PPI contributions. Non-participant observation combined with interviews offers the opportunity for a more detailed understanding of how PPI actually functions within trials [5, 27]. This paper aims to (1) describe how PPI functions in trial oversight, using multiple qualitative methods, and (2) contribute to recommendations for maximising PPI in clinical trials, in collaboration with public contributors with experience of involvement in trial oversight.

## Methods

Data presented were collected within a larger ethnographic study of the role and function of trial oversight committees and their contribution to trial conduct in trials facing challenges [29, 30]. We used a cross-sectional ethnographic study design, including a combination of non-participant observation of TSC and TMG meetings, and interviews with members of these committees and other key informants related to trial oversight. The methods have been reported in detail previously [29, 30] and are summarised here.

### Sampling

Data were collected between March 2013 and January 2014 from eight phase III UK trials identified as facing challenges (e.g. recruitment problems, protocol deviation or amendments). Trials were purposively sampled to represent a range of clinical topics, healthcare settings and type of intervention. Personnel from within these trials were also purposively selected to include differing roles and perspectives and included TSC and TMG Chairs, Chief Investigators (CIs), trial team members, trial sponsors, independent TSC members and patient or public contributors (henceforth referred to as ‘public contributors’). Additional key informants from outside the eight trials were also interviewed, including trial funders, a sponsor and senior researchers with experience of other challenging trials. Data collection and sampling continued until data saturation was reached (no new themes emerged during data analysis) [31].

### Data collection

For each trial, one TSC meeting and one TMG meeting in the study period were targeted for observation by the researcher. Detailed field notes were taken during and after the meetings, guided by an observation schedule [30]. Semi-structured interviews were conducted either face-to-face or by telephone, before or after the observed meetings, according to participant availability and preference. Topic guides were developed and used to guide interviews and included questions relating to PPI (Table 1) [29].

**Table 1:** Patient and Public Involvement related questioning within interviews*

**Questioning for trialists/funder representatives/sponsor representatives**	
Views of PPI and actual impact on trial conduct decisions	
Role of PPI on the TMG/TSC	
Background of public members on the TMG/TSC	
Reasons for selecting public members to be part of the TMG/TSC	
Views on which committee (TMG/TSC) public members are best placed to contribute	
**Questioning for public contributors**	
Experience in trial oversight committees	
Their understanding of their role in the committee	
Examples of how they perceived they had influenced trial conduct	
How they felt during oversight meetings	

*PPI* patient and public involvement, *TMG* trial management group, *TSC* trial steering committee

*Extracted from interview topic guides

Written informed consent was provided by all committee members and interview participants, in advance of each observation or interview. All observations and interviews were undertaken by one qualitative researcher (AD) and recorded and transcribed verbatim.

### Data analysis

Thematic analysis was used to identify themes in the observational data (meeting transcripts and field notes) and interview transcripts, using a mixture of inductive and deductive coding and techniques of constant comparison [32, 33], as previously described [29]. Data analysis was conducted alongside data collection to allow emergent themes to inform subsequent data collection. Coding was conducted by AD, with a subset of interview and meeting transcripts coded by other study members [29]. NVivo 10 [34] was used to aid organisation and refinement of codes. To inform this paper, a second stage of more fine-grained coding on data related to PPI was conducted by a second qualitative researcher (AN) after familiarisation with the dataset. Mind maps were used to aid conceptualisation and organisation, then codes were developed into broader categories and higher-level themes, which were discussed and agreed with AD, JAL, AS and KC. Finally, a descriptive account was written integrating both observational and interview data (KC, AN). Data was triangulated to explore areas of divergence and convergence in the two datasets, and the different participant perspectives represented.

Data presented have been anonymised to protect confidentiality. Quotes from interviews are labelled with participant ID and trial number. Observational data from meeting recordings and observation notes are labelled according to the type of meeting and trial number. Findings are reported using the Standards for Reporting Qualitative Research (SPQR) Checklist [35] (Supplementary table 1), and the GRIPP2 short-form checklist for reporting PPI in research (Supplementary table 2) [36].

### Methods used for PPI within the study

Two public contributors with experience of involvement in trial oversight committees (MEP and GT) were invited by JAL to contribute to the writing of this paper. Both accepted. Public contributors were not involved in the design of this study.

## Results

### PPI representation in eight trials facing challenges

Overall, 14 meetings (eight TSC, six TMG) were observed from the eight trials (Table 2), with details of response rates reported previously [29].

**Table 2:** Randomised trials facing challenges and public contributors

Trial number	Clinical area/population	Meetings observed		Public contributors at meeting		Discussion of PPI if no contributors at meeting observed		PPI in the trial reported by interviewees, if not observed
		TSC	TMG	TSC	TMG	TSC	TMG	
1	Oncology	✓	✓	0	0	x	x	On TMG, although not at meeting observed
2	Oncology	✓	x	0	–	x	–	On TMG.
3	Arthritis	✓	✓	0	0	x	✓	Previous involvement of a public member, trying to recruit a new member
4	Frailty	✓	✓	0	0	x	✓	Confusion as to whether certain trial interactions with patients counted as PPI. Patient charity representative invited to TSC meetings with poor attendance.
5	Oncology	✓	✓	0	2	x	n/a	
6	Urology	✓	x	1	–	n/a	–	
7	Psychology	✓	✓	1	1*	n/a	n/a	
8	Oncology	✓	✓	1*	1*	n/a	n/a	

*PPI* patient and public involvement, *TMG* trial management group, *TSC* trial steering committee, *n/a* not applicable, − not observed

*Public contributors interviewed. Different public contributors were present at the TMG and the TSC of both trials 7 and 8 for a total of 7 public contributors observed in meetings

Linked to these eight trials, 66 interviews were conducted with 52 individuals, including TSC and TMG members, three public contributors and other relevant informants (Table 3).

**Table 3:** Characteristics of interview participants

Participant ID	Role	Trial(s) involved in (subject area), where applicable
01	TSC Chair	1, 2 (oncology)
02*	Project lead	1, 5 (oncology)
03	TSC coordinator #1	1, 2 (oncology)
04	TSC coordinator #2	1, 2 (oncology)
05*	Sponsor representative	1, 2 (oncology)
06*	Sponsor representative	1, 2 (oncology)
07*	Trial manager	1 (oncology)
08*	CI	2 (oncology)
09*	Trial manager	2 (oncology)
10*	Trial manager	3 (arthritis)
11*	Statistician	3 (arthritis)
12*	Trial manager	3 (arthritis)
13	Statistician	3 (arthritis)
14	CI	3 (arthritis)
15	TSC Chair	3 (arthritis)
16*	Trial manager	4 (frailty)
17	TSC Chair	4 (frailty)
18	CI	4 (frailty)
19	TMG Chair	4 (frailty)
20*	TMG member	4 (frailty)
21*	Trial manager	5 (oncology)
22	Statistician	5 (oncology)
23*	CI	5 (oncology)
24*	Independent TSC member	5 (oncology)
25	Independent TSC member	5 (oncology)
26*	Trial manager	6 (urology)
27*	Trial manager	6 (urology)
28*	Statistician	6 (urology)
29*	CI	6 (urology)
30*	TSC Chair	6 (urology)
31*	Independent TSC member	6 (urology)
32*	TMG member	6 (urology)
33*	Trial manager	7 (psychology)
34*	CI	7 (psychology)
35*	Public contributor	7 (psychology)
36*	TSC Chair	7 (psychology)
37*	Statistician	7 (psychology)
38*	TSC Member	7 (psychology)
39	CTU Director	7 (psychology)
40*	Trial manager	7 (psychology)
41*	Trial manager	8 (oncology)
42*	Chair	8 (oncology)
43a*	Public contributor	8 (oncology)
43b*	Public contributor	8 (oncology)
44	Statistician	8 (oncology)
45*	Sponsor representative	8 (oncology)
46*	CI of other trial/member of TSCs	n/a
47*	Funder representative	n/a
48	Sponsor representative	n/a
49*	Funder representative	n/a
50*	Statistician	n/a
51*	Funder representative	n/a

*CI* Chief Investigator, *CTU* clinical trials unit, *TMG* trial management group, *TSC* trial steering committee

*Interviewee discussed PPI

Thirty-eight of the 52 participants discussed PPI across 43 interviews. Seven public contributors were present across three TSC and three TMG meetings within four of the eight trials (Table 2, trials 5–8). No public contributors were at observed meetings for the other four trials (trials 1–4), although interviewees reported that TMG membership included public contributors in two of these trials (1 and 2). In trial 3, interviewees described how a public member had contributed strongly at the trial start, but this had declined due to time commitments and they were actively trying to recruit a new member; there was brief discussion at the TMG meeting about obtaining public representation. For trial 4, an interviewee reported that a patient charity representative was invited to TSCs, but their attendance had been poor. The three public contributors interviewed were long-standing members of their respective groups (2 TMG, 1 TSC), had some experience of the disease/condition under study and/or experience of participating in previous oversight committees or patient advocacy groups. All had current or previous professional careers, two within an academic environment.

Eight themes relating to PPI in trial oversight were identified across two broader topics. Two themes relate to the role of PPI: (1) patient voice and advocacy and (2) extent of PPI role. Six themes describe barriers and facilitators to meaningful PPI in trial oversight: (1) confusion about definition and roles of PPI, (2) appreciated qualities of public contributors, (3) routes to invite public contributors, (4) involving PPI at the optimal level of oversight and trial stage, (5) formality of oversight meetings and technical language and (6) support and/or mentorship from the trial team.

### The role of PPI in trial oversight

#### Patient voice and advocacy

Trialists commonly referred to the role of public contributors in trial oversight as providing the patient perspective or voice, including patient advocacy. Public contributors were perceived as enabling the oversight committee to gain insight into participants’ experiences, views and preferences regarding the clinical condition and trial. They were also valued by trialists for their practical contributions, for example commenting on written documentation for trial participants. Patient advocacy included enabling links with appropriate patient networks and acting as a spokesperson in research settings outside of trial oversight, such as conferences or meetings.

…the reason why they [public contributor] were invited was so we got their views on how a patient would feel … [what they] might be going through, why patients might not want to participate in trials, and how … they think a [specialist clinician] should be approaching patients and sensitive issues. [07, Trial manager, trial 1]

The public contributors interviewed generally shared these perspectives, seeing their role as involving “quality control” and/or safeguarding to ensure the trial is conducted in a way that takes account of patients’ interests, does not cause harm, and produces outcomes that will benefit patients now and in the future.

My actual role, as I see it, is to represent the patients … to make sure their interests are dealt with correctly, that they are not just used as pawns in the research … as a piece of research data. At the end of it, it is a patient...a person that they are talking about, not just a source of data they can stick a tack on or switch on. [35, public contributor, trial 7]

However, a dissenting view was expressed by one public contributor, who felt that the advocacy role was better served by clinicians who can understand complex mechanisms underpinning a condition.

The idea of patient involvement in this [trial] seems to stem from the fact that they thought that maybe the doctors … would have their own ideals on what should be examined, which may not necessarily be that which benefits the patient the most. I don’t think the patient representatives are in a terribly strong position to be able to judge what does benefit the patients most in practical terms … the patients simply don’t know enough … understanding all the mechanism, it’s a very highly complex thing [43b, public contributor, trial 8]

#### Extent of PPI role

During meetings, public contributors were observed to contribute to discussions about recruitment issues and methods to improve the collection of patient-reported outcomes (observation notes, trial 8 TMG and TSC).

Trial manager, trial 8 TSC: …the trial is very dependent on questionnaires and quality of life outcomes. With it being one of our primary outcomes, it’s really important that we get the questionnaires back, and so if we can make that clearer to patients up front, so that they know that the trial involves a lot of questionnaires, then –

Public contributor, trial 8 TSC: Although you need not to put too much stress on it…. it’s a balance…I think you need to make them feel that any answer is useful rather than just abandoning the whole thing, yes.

In addition to this direct input into trial decisions, public members also sought clarification and/or commented on discussions related to data collection (trial 7 TMG and TSC, trial 8 TMG and TSC), protocol amendments (trial 5 TMG, trial 8 TMG), recruitment (trial 8 TMG), retention (trial 7 TSC), data analysis (trial 8 TMG), trial progression (trial 8 TMG) and provided encouragement/positive feedback to trial team members (trial 8 TSC).

Public contributor, trial 5 TMG: Can I just go back on what you said, it would be introduced through all arms of the trial?

CI, trial 5 TMG: Sorry that was me not speaking clearly…I didn’t mean all arms, I meant all stages.

All public contributors interviewed described their input to the improvement of the trial in some way, either through shared decision-making during meetings, or providing feedback on material behind the scenes.

…I did help to influence the decision, because my actual inputs were taken in and were used, along with everybody else’s. If you like, I completed the equation. [35, public contributor, trial 7]

However, some interviewees described public contributors as passive, endorsing decisions made by the rest of the committee.

…I don’t think they [public contributor] necessarily were involved in making the decision…I think they ratified our decisions rather than helped us make them, if that makes sense? [36, TSC chair, trial 7]

This more passive PPI role was also observed at three out of the six meetings where public members were present (observation notes, trial 5 TMG, trial 6 TSC, trial 7 TSC). Public contributors were not particularly vocal during these meetings, nor were they encouraged to express their views by other members of the trial team.

### Barriers and facilitators to meaningful PPI in trial oversight

Interviewees reflected on challenges to engaging PPI in trial oversight committees, as well as how to facilitate more meaningful ongoing involvement of public contributors. This included confusion around the definition and role of PPI, appreciated qualities of public contributors, routes to invite public contributors, involving public contributors at the optimal level of trial oversight and trial stage, formality of oversight meetings and technical language and support and/or mentorship from the trial team.

#### Confusion about definition and roles of PPI

Some trialists expressed ignorance or confusion about the active role that public contributors could play in trial oversight committees. At trial 4’s TMG meeting, there was uncertainty about which activities satisfied the label of PPI from the funder’s perspective. The distinction between qualitative research, stakeholder engagement and PPI activities was not well understood or delineated:

I think it’s perhaps recognising that tension between something that is very overtly and officially PPI…having lay members in your Trial Steering Committee or, “We had engagement with them when we were putting in the bid … and we did get people to look at the participant information sheets.”…but whether we were counting things, or whether we were saying it was the qualitative research element in the intervention development is a bit grey. [19, TMG Chair, trial 4 - TMG meeting]

Other trialists expressed a degree of reservation and scepticism about PPI in trial oversight and concerns that PPI could be tokenistic, motivated by funders’ requirements.

I agree it’s important, but the way it’s done at the moment, I think it’s hit and miss and often it’s tokenism, which is the worst of all worlds...I just wish it wasn’t like the Emperor’s new clothes, that people would have the courage to say “We don't think we need PPI at the moment, but when we do, we’ll get someone”…everybody at the moment thinks it’s fashionable, it’s what happens…I think there’s a great resource out there to get used, but I’m not sure of the optimal way of doing it [28, Statistician, trial 6]

#### Appreciated qualities of public contributors

The importance of selecting the “right” people to act as public contributors in trial oversight was seen as critical in moving towards an active PPI role.

…you need to find the right person because you don’t want it to just be tokenism… [31, Independent TSC member, trial 6]

However, selecting appropriate public contributors was challenging; one participant described it as “the biggest art form” [38, TSC member, trial 7]. Interviewees felt that previous committee or group experience (e.g. patient groups, ethics or other research committees) and an understanding of the research process could be beneficial. Some participants, including public contributors, felt that detailed knowledge of the research process, trial methodology and technical language was needed to enable input in some discussions.

…the issue I think we have with PPI is finding knowledgeable people who have an interest and understanding of research…sometimes it’s more of a hindrance because people don’t understand the need for research or the research processes [26, Trial manager, trial 6]

However, the value of “professionalised” public contributors with research experience and skills, was contested. While such detailed knowledge could be helpful, these “expert” public contributors were also perceived as potentially less representative of the “average” patient voice. Some trialists felt public contributors with a research and non-research background could keep a “healthy perspective about the trial” [21, Trial manager, trial 5]*.*

Some interviewees also reflected on instances when a public contributor’s experience and/or organisational affiliations created a personal agenda or an “axe to grind” [28, Statistician, trial 6], driving their desire to be involved in trial oversight. In these instances, PPI was perceived to be misguided and unhelpful in facilitating effective trial oversight as these contributors were not focused on the trial purpose or validity and discussions were considered off task.

We had one example where…the patient representative was from the [national organisation]. They have very definite opinions and beliefs about [topic], and basically this representative just didn’t believe the trial was necessary…So that was a bad experience. Where I don’t think that person was in any way, shape or form, protecting the safety of the participants…Nor was it protecting the integrity of the trial. [37, Statistician, trial 7]

Conversely, the absence of a “personal agenda” together with direct experience of a relevant clinical condition was valued in public contributors.

Interviewer: How do you know she’s a good patient advocate?

[42, Chair, trial 8]: firstly…a breadth of knowledge which comes across in the way she responds to comments and questions – sound stuff…she’s sympathetic, empathetic and…very well focused…Also…she has cared for somebody who had [clinical condition], so there’s an inside knowledge, a personal aspect to her as well which she doesn’t let come through at the meetings. There’s no personal agenda.

However, some trialists noted direct experience of a disease or condition could at times make it difficult for a public contributor to regularly attend meetings. For example, in the event of relapse of a condition or the physical limitations of an on-going condition. Other participants reported that public contributors’ broader commitments could also impact on continued involvement.

The lay reps come on board early. They are enthusiastic. They then do not come to any more meetings…I think that is an incredible shame, if they just decide that it is not for them anymore, or they are too busy, or if they are a lay rep because they have got a condition, and they get ill. So I think, to me, choosing the lay representatives is the biggest challenge. [38, TSC member, trial 7]

#### Routes to invite public contributors

The presence of more than one public contributor in trial oversight committees was suggested to be helpful as their shared experience could facilitate self-confidence in their role, and hence contribution to the discussions. Having two public contributors was also seen to help with continuity, by increasing the likelihood that at least one would be able to attend meetings.

Interviewer: …and you have two because...?

02, Project lead, trials 1,5: Because of continuity, partly people can’t always turn up to meetings, partly because when you’ve got a lot of high powered people around a table it can be difficult for people to make their voices and opinions heard and…two people there can in fact really help, partly through if they do have a spell of ill health they don’t feel obliged to make an effort to engage when they don’t feel up to it…

Methods used by trialists to invite public contributors to join oversight committees included contacts such as friends or colleagues, existing networks or committees and direct advertising. In some trials, these methods were applied opportunistically whereas in others more structured or formal efforts were made to invite public contributors. Trialists did not offer views on the method they thought most effective, rather they described examples of when their chosen process had proved effective or not. In one trial, a public member had moved from the iDMC to the TSC after the decision was taken that the iDMC could stand down; this was viewed positively as the contributor came with assumed levels of knowledge and understanding about the trial.

…she transferred from the iDMC…We had the patient representative, she had to stand down, and then when the decision was taken, that the DMC could stand down, she was a kind of gift-wrapped transfer. [37, Statistician, trial 7]

The CI of another trial described how he had recruited a public contributor opportunistically through another institution’s PPI network; however, difficulties had arisen due to the contributor’s ill health.

He [public contributor] was given to me by somebody else. I said can you find me a lay person from your pool of PPI people...it's another university where they've got a very active and developed PPI network…and they're [public contributor] currently unwell actually. So that's a problem. [46, CI of other trials/TSCs member]

Participants differed as to whether they felt advertising was an appropriate way to engage effective public contributors. One interviewee felt you would be likely to get someone with a personal agenda, who would not necessarily participate for the right reasons.

It’s actually quite hard to find them [public contributors] and if you advertise you tend to get people with an agenda. They get involved because it's something they want to have a go about…They're not always there for good purposes... [29, CI, trial 6]

However, a trial manager described a positive experience advertising and obtaining a public contributor who had previous experience through a relevant research network, and this had been helpful.

…we asked for the help of the [research network]…and they went through the involvement centre to get expressions of interest from people who had…done some involvement work previously…[public contributor]’s good…been helpful. [33, Trial manager, trial 7]

#### Involving PPI at optimal level of oversight and trial stage

Participants were asked on which oversight committee they thought public contributors were best placed to contribute. Some reported they did not have a strong opinion either way and/or enough experience to judge which level of oversight would be optimal. Most participants that did have a view felt that PPI was most relevant at the TMG level where the details of a trial are resolved, for example, patient information leaflets and questionnaires.

I think probably TMG…the best place to get input from them because that's really where we make decisions about changes to questionnaires and things like that... [29, CI, trial 6]

One senior trialist reflected in more depth on the rationale behind including PPI at the TMG and not the TSC level; exposure to sensitive data and discussions that may take place at the TSC could impact adversely on public contributors’ future contacts with patients and/or clinicians. In addition, this participant felt that the PPI patient advocate role could be problematic for the TSC with its focus on “independent” monitoring and oversight, which may preclude public contributors with strongly held views about particular treatments.

…we decided the TSC…wasn’t quite the right place to put patient representatives…it could cause problems for them, the trial, the patients we have are…very often advocates for (the) treatment of the research - we thought…the discussions that they have with patients and doctors, they could find themselves in a more difficult position by having been exposed to certain data and conversations and so we thought the TMG is probably the right place... [02, Project lead, trials 1,5]

PPI contributions at the TMG level were also thought to be better due to the greater frequency of these meetings, which could better facilitate the capacity of PPI to impact on trial conduct. However, some trialists noted that more meetings required a greater time commitment from public contributors, which might influence their ongoing engagement and involvement. The infrequency of TSC meetings could also reduce the workload for public contributors and help their engagement. For example, in one trial, it was deemed more appropriate for public contributors to join the TSC as it was felt the patient population were unlikely to cope with the heavy workload and commitment of the TMG.

…because we look at people who are pretty old and frail, it’s actually quite hard to do that…in the TMG because that’s a group where you’re asking quite a lot of people to come along to a monthly meeting to read stuff, to really get very immersed in the whole thing and to potentially have to do stuff as well… [31, Independent TSC member, trial 6]

There was also discussion about PPI that was carried out separately to oversight meetings. As previously described for trial 4, there was confusion at the TMG meeting about what “counted” as PPI. An interviewee from the trial described leading a patient group which met separately to TMGs, reviewed study documentation and was planning to conduct training for new researchers. She considered this to be the PPI for the trial.

Interviewer: …as I was listening to you, I just thought, “Well, gosh, isn’t that…PPI involvement?”

20, TMG member, trial 4: I don’t know. It’s a horrible boundary, isn’t it?...I’m in charge of PPI. So, we’ve got this PPI group and we meet in between TMGs and they’ve been doing loads of work on all the documentation and they’re going to do training, when we get our new Research Associate…It just feels like that’s the PPI.

Other participants felt the level of oversight was less important than the stage of the research process and from a funder’s perspective, the early stages of trial design and development of the grant application were crucial stages for PPI to help ensure feasibility.

I don’t mind where they are really as long as they have some role, preferably at the time when the trial is being designed because I think it’s really important they have their input into the feasibility... [51, Funder representative]

#### The formality of oversight meetings and technical language

Participants reported that the formality of oversight meetings could hinder the engagement of public contributors who may be unaware of implicit “rules of engagement” in academic settings.

…the patient representative had some issues with maintaining her attendance at the meetings...I suspect that the strangeness of…being on a TSC, because it is a fairly unusual activity. I think we tend to forget that, as experienced researchers. But it’s got a lot of rules, it’s quite formal; it’s designed for academics really, to give their opinions. I think she found the whole process bewildering. [37, Statistician, trial 7]

Additionally, technical language and jargon used in oversight meetings could affect public contributors’ understanding of topics discussed, and hence impact on the extent of their contributions.

It’s okay for me now, but I didn’t actually ask them when I first started because I was a little shy…but that became embarrassing, because they’d been talking about it so long, I didn’t like to say, “Just a minute, what does that mean?” [43b, public contributor, trial 8]

During two meetings observed, senior trialists checked public members’ understanding of topics and provided additional explanation to aid understanding (observation notes, trial #5 TMG, trial #8 TSC). A trial sponsor suggested that advance planning was needed to ensure that statistical issues were presented in a way that was more accessible to committee members without statistical expertise:

I think the issues that come up from an iDMC need to be very, very clearly laid out in advance by the relevant statisticians and framed in the way that a savvy clinician or lay member… - and I think lay people probably should be involved - the statistician needs to set up the presentation in such a way that it doesn’t take statistical skills to follow. [06, Sponsor representative, trials 1&2]

#### Support and/or mentorship from the trial team

Some interviewees discussed training and/or mentoring of public contributors to facilitate their sense of inclusion and contribution to meetings.

...we make sure that any patient representative has adequate training at the beginning before joining so we have training teleconferences and just taking them through the protocol and what it means and the organisation of the TMG… [21, Trial manager, trial 5]

The public contributors interviewed valued support they had received in the form of email or telephone contact with team members. This informal, ad hoc support mechanism, which allowed for PPI input outside of oversight meetings, was seen as important.

I read it [notes of meetings] as well. If I can’t understand it, I email [trial manager] and say, “Look, I can’t understand this. What is it we are trying to say here? What is trying to be said?” [35, public contributor, trial 7]

In some instances, concerted efforts were made by trial team members to facilitate ongoing PPI outside of meetings and build relationships with public contributors. For example, contributors were encouraged to contact a specific trial team member if they wanted to discuss anything they were concerned about or did not fully understand. The trial manager at one TMG meeting mentioned a follow-up meeting with the public contributors to ensure clarity of discussions.

21, Trial manager, trial 5 TMG: [public contributor 1] and [public contributor 2] I am conscious that was a quite full of items agenda. I would like to follow this up with you, tomorrow or in the next couple of days, in a separate teleconference or in e-mail, so that everything is clear to both of you. Particularly [public contributor 1] as you just joined the TMG.

Public member, trial 5 TMG: Yes, sounds fine.

One trial manager described how their trials unit had a PPI support staff member:

…we’ve actually as a CTU changed our approach…we’ve now got somebody to…act as PPI support…who is employed by the CTU…they [public contributors] have somebody that they can go to, to ask questions… [12, Trial manager, trial 3]

Some interviewees also described the importance of the Chair in facilitating PPI contributions during meetings, particularly for those public members who were less confident at speaking up in meetings.

…I think you need to either select people who are confident about speaking in those kind of circumstances or you need to have a TSC chair who will drag views out of them to specifically ask them what they think and for their contribution…really the TSC chair needs to be specifically reminded that they have to involve the public members. [50, Statistician]

Conversely, in one TMG, a lot of cross-talking between members was observed, with the Chair seeming less effective at running the meeting (observation notes, trial 8 TMG). The public contributor who was interviewed subsequently commented that the PPI update at the TMG was completely overlooked and that timekeeping was an ongoing issue:

I felt…the meeting was a bit…lop-sided. We went through the last agenda items as if they weren’t there, including the patient representative’s report… [Chair] didn’t even mention it...But, the time was pressing and…[Chair] didn’t quite keep to time. But, that’s been a general feature of those TMGs…sometimes, it’s gone on for much longer than two hours. [43b, public contributor, trial 8]

### Influence of PPI on study results

MEP and GT commented on the rigour of the research and the credibility of the results, commenting that many of the findings were similar to their own experiences in trial oversight committees, and also made suggestions to improve clarity of the results.

## Discussion

To our knowledge, this is the first multi-method qualitative study to investigate PPI activity within trial oversight committees. Data were collected from observations of TSC and TMG meetings across eight clinical trials facing challenges, and interviews with key stakeholders including a wide range of trial staff, and public contributors. PPI was generally regarded as beneficial to trials, providing the patient voice and/or playing a patient advocacy role. Examples of active and valued PPI input were identified; however, not all trials had current public contributors. Concerns around tokenism were raised, as well as confusion about PPI. We describe facilitators to maximise meaningful PPI input in trial oversight. These include the need for careful consideration and planning on how to recruit public contributors, the optimal level of trial oversight and/or trial stage(s) to involve public contributors and their ongoing support/mentoring needs.

Our study found that public contributors were not always active in meetings observed. Taken at face value, this may lead us to believe that the public contributor had no impact on trial decisions; however, Crocker et al. reported that this “passive presence” type of public contributor could still impact the way professionals think simply by being present at research meetings [27]. Our findings demonstrate uncertainty as to how to undertake PPI effectively with tokenistic PPI raised as a concern. Previous research has identified tokenistic PPI as a problem in clinical trials and other studies, where it has been described as a “self-fulfilling prophecy” or a “self-perpetuating cycle” [5, 19–21, 23, 24]. A lack of clarity or understanding about PPI leads to it being carried out in a tokenistic way that allows for little impact on the research. Researchers thus conclude that PPI has little value and the cycle continues [5, 23]. Some trial committee members interviewed expressed rather counter-productive views about public members, for example, suggesting that public contributors may not make much of an effort when they are ill. For PPI to have a meaningful role in trial oversight, all trial team members need to develop a shared view of the nature and role of PPI. This includes an emphasis on co-production with a shift towards a more equal relationship of power between public contributors and other trial team members, which may be uncomfortable for some researchers [37, 38].

We found that using technical language during meetings could exclude public contributors, as has been reported in other studies [4, 21, 24, 28, 39]. Participants highlighted the need to present and discuss complicated technical matters (such as statistics) in a more “lay” and engaging way during oversight meetings. Interviewees in our study also described the need for good chairing skills to ensure all voices are heard in meetings, including public members. The importance of the Chair in ensuring patient understanding and inclusion at research meetings has also recently been highlighted in the Cancer Research UK PPI toolkit [40]. Practical guidance for Chairs of research meetings is available and highlights the importance of developing relationships with public members and fostering regular communication between meetings [41]. Similarly, participants in our study described how support and/or mentorship for public contributors could facilitate contributors’ input and sense of inclusion within oversight meetings. This included induction meetings to explain the trial protocol and PPI roles and regular communication and support between oversight meetings to clarify issues. Previous studies have also reported the benefit of informal and ad hoc support provided by the research team [4, 5, 24, 28, 39], and Bagley et al. have developed resources for providing clear information about trial oversight to public members including their role [16].

Interviewees also described uncertainty in how to go about selecting public contributors, with one describing it as an “art form”. The importance of finding the “right” public contributors has been highlighted, with valued characteristics reported to include enthusiasm, confidence, impartiality and an interest in and understanding of research [4, 28, 39]. However, we also identified concerns that “professionalised” public members, with previous research and/or committee experience, may not represent the “average” person within the relevant population. These “professionalised” members may be more likely to be selected by researchers for public roles as they fit more easily into academic processes, which better suit researchers’ agendas [39, 42]. Staley, however, asserts that public contributors do not lose their lay status by virtue of their involvement in research [43]. She argues that certain aspects of a research project may benefit from the perspectives of public contributors with more research experience (e.g. the technical aspects of trial design), whereas contributors without this experience may be ideally suited to other aspects (e.g. perspectives on recruitment and patient-facing materials). It is important that there is clarity about roles and responsibilities of public contributors, including adequate support tailored to each role [44–46].

Rather than focusing on finding public members who are “representative”, INVOLVE recommends seeking people with the range of perspectives needed to inform the research, as a few public members can never be representative of the entire population of interest [44, 47]. Interviewees in our study recommended the inclusion of experienced and less experienced public members to provide a mix of skills and experience, help to build confidence in public members and help with continuity, similar to findings from the EPIC study, and as recommended by INVOLVE [39, 44]. A recent qualitative study investigating PPI in surgical trials reported that a “two-tier” PPI model whereby a larger panel of public contributors are consulted intermittently, with one or two of the contributors more regularly involved with the trial team, could lead to better representation and engagement [22]. Our study also found that there is no one best avenue to recruit public contributors; this depends on the trial and patient population, and adequate consideration should be given to this at trial design stage [44]. Improving diversity of PPI is also reflected in the NIHR National Standards for PPI in research [48].

Our finding that public contributors may be best placed in a TMG rather than solely a TSC supports findings from the EPIC study [5]; however, our findings highlight that this is dependent partially on the trial. EPIC found that interviewees from trials which included PPI solely on their TSCs (with no other PPI input) were less likely to report impactful PPI, with TMG involvement and other forms of more responsive PPI reported to foster more meaningful involvement [5]. One trialist in our study felt the TSC was more appropriate for PPI due to the study population being unlikely to cope with the greater frequency of TMG meetings. However, the actual level of PPI within this trial is uncertain; the public contributor was not observed to be particularly active during the TSC meeting, and trialists from this trial expressed reservations about tokenistic PPI. Previous research supports the need to adapt PPI to the emergent needs of a trial in order to facilitate more impactful PPI [5, 20, 28]. For some trials and patient populations, more “responsive”, flexible modes of PPI may be more appropriate, for example holding separate PPI meetings at times and locations accessible to public contributors, and/or seeking PPI on an “as needed” basis, rather than viewing oversight meetings as the only format for PPI [5, 28]. Early PPI at trial design stage was raised as especially important from the perspective of funders in our study and has been reported to be associated with greater PPI impact in trials [5, 20, 21, 28].

A key strength of this study is that non-participant observation of oversight meetings was combined with interviews with key stakeholders in ongoing trials to gain a greater understanding of how PPI actually functions in trial oversight. This allowed an in-depth exploration from different perspectives that would not have been possible using either method alone. Our observational data revealed how public contributors varied in their inputs into discussions at oversight meetings and the role of the Chair in facilitating public contributions. A limitation of this study is that these eight trials are not representative of all late-phase randomised trials—all were UK-based and publicly-funded. Trials conducted in other countries, with other funders and alternative oversight structures, may experience different issues. Additionally, only trials undergoing challenges were included. Whether PPI functions differently in trials that are undergoing challenges compared with those that are not merits further research. The selection of trials undergoing challenges ensured that these oversight meetings were critical to trial progress, so it was notable that public contributors were present at only six of the 14 observed meetings. At two additional meetings (one TMG, one TSC) public contributors were invited but not in attendance. This study focused on the role and function of trial oversight committees in general rather than PPI specifically; however, this could also be seen as a strength as it may have reduced the likelihood of “social desirability” issues as identified by one interviewee in describing PPI as “the emperor’s new clothes”. Public contributors were not involved in the design of this study; however, they provided a valuable perspective, commenting on the integrity of the results and helping to shape the recommendations in this paper. Although only three public contributors were interviewed, all interviewees approached agreed to be interviewed. However, the majority of interviewees discussed PPI and we were able to observe seven public contributors ‘in action’ during meetings. Future research would benefit from a larger number of interviews with public members to enable a more in-depth comparison of their perspectives with other trial team members.

Based on our study and previous literature, we provide practical suggestions on how to maximise meaningful PPI input in trial oversight (Table 4).

**Table 4:** Recommendations: Enhancing patient and public involvement in trial oversight

Steps to maximise the benefit of PPI	Facilitated by:
Agree PPI needs of trial with research team (early trial design stage)* • Define clear role/s and/or goals for PPI • Define trial stages that PPI needed – including trial design stage • Define demographic characteristics, skills and experience desired from public contributors • Identify optimal format for PPI (e.g. attendance at trial oversight committee(s), and/or separate PPI meetings, email/telephone) – negotiate this with public contributors recruited • Ensure PPI is costed properly to re-imburse for public contributors’ time and expenses, including carer support if appropriate • Ensure PPI plan is fully justified in both funding applications and trial protocols	• Training for research team on the role PPI can play in trials and how to maximise meaningful input. Include opportunities for discussion. • Accessing useful PPI planning resources (e.g. PPI Toolkit for clinical trials [16]), and the NIHR INVOLVE public involvement cost calculator (https://www.invo.org.uk/resource-centre/payment-and-recognition-for-public-involvement/involvement-cost-calculator/) • Consulting with existing patient or public panels may be useful at this early stage to help define PPI needs • Consider inviting public contributors to be co-applicants on grant applications
**Recruit diverse public contributors** • Consider effective routes to invite public contributors (e.g. local groups, national organisations, existing PPI groups, advertising) to allow for diversity • Recruit more than one public contributor, focusing on diversity of characteristics, skills and experience (e.g. some with research/committee experience and some without)	• Drawing on insight of colleagues with previous experience of identifying and working with public contributors from target population, and existing relevant patient or public panels.
**Engage public contributors in trial oversight** • Provide an induction to the trial for public contributors including oversight processes • Ensure meeting Chairs have skills to engage public contributors • Provide on-going mentoring/support to public contributors in between oversight meetings, e.g. regular meetings/phone calls to foster relationship, answer queries, and provide training as deemed appropriate • Accommodate needs of the patient group to facilitate PPI attendance at meetings (e.g. shorter meetings/attendance for part of the meeting, care needs or caring responsibilities) • Adapt format of PPI to the emerging needs of the trial and public contributors	• Accessing PPI training/support for trial team including meeting Chairs (e.g. UKCRC/NCRI PPI in research groups - Guidance for Chairs [41]) • Identifying appropriate research team member to provide on-going support to public contributors, and allowing protected time for this within their role • Drawing on relevant resources provided in the PPI Toolkit for clinical trials [16] to familiarise public contributors with their role in the trial oversight committee, academic terminology and environment • Considering both academic and public contributors’ commitments

*PPI* patient and public involvement

*Relevant patients or lay members of the public should be involved in defining the PPI needs for a trial

These include providing support and/or training for the trial team in how to undertake PPI effectively (including how to support and engage public contributors during and in-between oversight meetings), early planning to establish clear goals and plans for PPI within a trial, adapting oversight processes to facilitate effective PPI (especially PPI by “non-professional” members), adjusting the format of PPI to the emerging needs of the trial and regular support for public contributors from the trial team. Our findings highlight the need for further research into how to adapt PPI to the needs of individual trials, rather than adopting a prescriptive model, and this was recently prioritised as a key area for research from a Delphi survey of PPI stakeholders [20]. Future research should also seek to develop methods to improve the inclusion of “non-professional” public contributors within trials and to evaluate the impact of PPI training/support for both trialists and public contributors.

## Conclusions

To facilitate more meaningful PPI within clinical trials, it is essential that that PPI planning happens at early trial design stage. This includes consideration of how to recruit and involve public contributors within trial oversight, as well as support and mentorship for both public contributors and trialists in how to engage with PPI effectively to maximise trial benefit. Relevant patients and/or members of the public should be involved in the planning process, and trial teams may wish to consider engaging existing patient or public panels to facilitate this.

### Influence of PPI on study discussion and conclusions

Public contributors (MEP and GT) provided valuable feedback on topics that should be brought out more strongly in the Discussion, Conclusions and the recommendations provided in Table 4, as well as suggestions to improve the clarity of the recommendations presented.

## Supplementary information

**Additional file 1: Supplementary table 1.** Standards for Reporting Qualitative Research (SPQR) Checklist.

**Additional file 2: Supplementary table 2.** Gripp-2 short form checklist.

## Data Availability

The datasets generated and analysed during the current study are not publicly available due to the nature of the consent provided by participants, but anonymised data are available from the corresponding author on reasonable request.

## Abbreviations

CI: Chief Investigator
iDMC: Independent Data Monitoring Committee
MRC: Medical Research Council
NIHR: National Institute for Health Research
PPI: Patient and Public Involvement
TMG: Trial Management Group
TSC: Trial Steering Committee
