# Supplementary material for: Understanding and optimising patient and public involvement in trial oversight: an ethnographic study of eight clinical trials
Source: Trials. 2020 Jun 18;21:543. doi: 10.1186/s13063-020-04495-9 (PMC7302397; doi:10.1186/s13063-020-04495-9)
Supplement: Supplementary file 1 — Additional file 1: Supplementary table 1. Standards for Reporting Qualitative Research (SPQR) Checklist. [file 13063_2020_4495_MOESM1_ESM.docx]

**Supplementary table 1: Standards for Reporting Qualitative Research (SPQR) Checklist (1)**

| **No.** | **Topic** | **Item** | **Location in manuscript (lines)** |
| --- | --- | --- | --- |
| **Title and abstract** | | | |
| S1 | Title | Concise description of the nature and topic of the study. Identifying the study as qualitative or indicating the approach (e.g., ethnography, grounded theory) or data collection methods (e.g., interview, focus group) is recommended | 1-2 |
| S2 | Abstract | Summary of key elements of the study using the abstract format of the intended publication; typically includes background, purpose, methods, results, and conclusions- | 22-50 |
| **Introduction** | | | |
| S3 | Problem formulation | Description and significance of the problem/phenomenon studied; review of relevant theory and empirical work; problem statement | 55-92 |
| S4 | Purpose or research question | Purpose of the study and specific objectives or questions | 92-95 |
| **Methods** | | | |
| S5 | Qualitative approach and research paradigm | Qualitative approach (e.g., ethnography, grounded theory, case study, phenomenology, narrative research) and guiding theory if appropriate; identifying the research paradigm (e.g., postpositivist, constructivist/interpretivist) is also recommended; rationale^b^ | 97-102 and research paradigm described in our earlier publication (2) |
| S6 | Researcher characteristics and reflexivity | Researchers’ characteristics that may influence the research, including personal attributes, qualifications/experience, relationship with participants, assumptions, and/or presuppositions; potential or actual interaction between researchers’ characteristics and the research questions, approach, methods, results, and/or transferability | Described in our earlier publication (2) |
| S7 | Context | Setting/site and salient contextual factors; rationale^b^ | 97-99, 104-105, and our earlier publication (2) |
| S8 | Sampling strategy | How and why research participants, documents, or events were selected; criteria for deciding when no further sampling was necessary (e.g., sampling saturation); rationale^b^ | 104-114, and our earlier publication (2) |
| S9 | Ethical issues pertaining to human subjects | Documentation of approval by an appropriate ethics review board and participant consent, or explanation for lack thereof; other confidentiality and data security issues | 128-129, 677-681 |
| S10 | Data collection methods | Types of data collected;  details of data collection procedures  including (as appropriate)  start and stop dates of data collection and analysis,  iterative process,  triangulation of sources/methods, and  modification of procedures in response to evolving study findings;  rationale^b^ | 99-100  115-121, 128-131  104  112-114, 135-137  143-146  112-114, 135-137  Described in our earlier publication (2) |
| S11 | Data collection instruments and technologies | Description of instruments (e.g., interview guides, questionnaires) and devices (e.g., audio recorders) used for data collection; if/how the instrument(s) changed over the course of the study | 117-121, 129-131, Table 1 |
| S12 | Units of study | Number and relevant characteristics of participants, documents, or events included in the study; level of participation (could be reported in results) | 104-112, 159-167, Tables 2, 3 |
| S13 | Data processing | Methods for processing data prior to and during analysis, including  transcription,  data entry, data management and security,  verification of data integrity,  data coding, and  anonymization/deidentification of excerpts | 129-131,  138-139, 689-691  137-138, 520-522  137-141,  147-150, 685-686  and our earlier publication (2) |
| S14 | Data analysis | Process by which inferences, themes, etc., were identified and developed, including the researchers involved in data analysis; usually references a specific paradigm or approach; rationale^b^ | 128-141, 661-670, and our earlier publication (2) |
| S15 | Techniques to enhance trustworthiness | Techniques to enhance trustworthiness and credibility of data analysis (e.g., member checking, audit trail, triangulation); rationale^b^ | 137-146, and our earlier publication (2) |
| **Results/findings** | | | |
| S16 | Synthesis and interpretation | Main findings (e.g., interpretations, inferences, and themes); might include development of a theory or model, or integration with prior research or theory | 179-518 |
| S17 | Links to empirical data | Evidence (e.g., quotes, field notes, text excerpts, photographs) to substantiate analytic findings | 196-518 |
| **Discussion** | | | |
| S18 | Integration with prior work, implications,  transferability, and contribution(s) to the field | Short summary of main findings;  explanation of how findings and conclusions connect to, support, elaborate on, or challenge conclusions of earlier scholarship;  discussion of scope of application/ generalizability;  identification of unique contribution(s) to scholarship in a discipline or field | 524-534  535-615  622-627  642-655, Table 4 |
| S19 | Limitations | Trustworthiness and limitations of findings |  |
| **Other** | | | |
| S20 | Conflicts of interest | Potential sources of influence or perceived influence on study conduct and conclusions; how these were managed | 694-695 |
| S21 | Funding | Sources of funding and other support; role of funders in data collection, interpretation, and reporting | 696-701 |

^b^The rationale should briefly discuss the justification for choosing that theory, approach, method, or technique rather than other options available, the assumptions and limitations implicit in those choices, and how those choices influence study conclusions and transferability. As appropriate, the rationale for several items might be discussed together.

1. O'Brien BC, Harris IB, Beckman TJ, Reed DA, Cook DA. Standards for reporting qualitative research: a synthesis of recommendations. Academic medicine : journal of the Association of American Medical Colleges. 2014;89(9):1245-51.

2. Daykin A, Selman LE, Cramer H, McCann S, Shorter GW, Sydes MR, et al. What are the roles and valued attributes of a Trial Steering Committee? Ethnographic study of eight clinical trials facing challenges. Trials. 2016;17(1):307.
