# Supplementary material for: Understanding and optimising patient and public involvement in trial oversight: an ethnographic study of eight clinical trials
Source: Trials. 2020 Jun 18;21:543. doi: 10.1186/s13063-020-04495-9 (PMC7302397; doi:10.1186/s13063-020-04495-9)
Supplement: Supplementary file 2 — Additional file 2: Supplementary table 2. Gripp-2 short form checklist. [file 13063_2020_4495_MOESM2_ESM.docx]

**Supplementary table 2: Gripp-2 short form checklist (1)**

| Section and topic | Item | Reported on page No |
| --- | --- | --- |
| 1: Aim | Report the aim of PPI in the study | 4 |
| 2: Methods | Provide a clear description of the methods used for PPI in the study, including both positive and negative outcomes | 8 |
| 3: Study results | Outcomes – Comment on the extent to which PPI influenced the study overall. Describe positive and negative effects | 23 |
| 4: Discussion and conclusions | Outcomes – Comment on the extent to which PPI influenced the study overall. Describe positive and negative effects | 29 |
| 5: Reflections/critical perspective | Comment critically on the study, reflecting on the things that went well and those that did not, so others can learn from this experience | 28 |

PPI=patient and public involvement

1. Staniszewska S, Brett J, Simera I, Seers K, Mockford C, Goodlad S, et al. GRIPP2 reporting checklists: tools to improve reporting of patient and public involvement in research. BMJ. 2017;358:j3453.
